# Demystifying cognitive science: explaining cognition through network-based modeling

**DOI:** 10.3389/fphys.2015.00088

**Published:** 2015-03-19

**Authors:** Emma K. Soberano, Damian G. Kelty-Stephen

**Affiliations:** Grinnell CollegeGrinnell, IA, USA

**Keywords:** cognitive science, network analysis, vector autoregression, fractal, multifractal, perception

Network science has made what once seemed miracle into practical science: we can set up conditions for self-building systems, and we can use their patterns of variability to identify and explain processes underlying prospective control in biological systems.

Behavior lifts itself from a sprawling mass of random events and directs itself prospectively toward events in the future not existing presently except as possibilities (Turvey, 1992). Possible effects become causes for current action. This control balks ordinary cause-and-effect sequences: organisms move themselves to seek stimuli which may never exist. Other sciences must wait for outside forces to stimulate a system into action, but cognitive science has begged your patience for a central article of faith: if you accept that a system can steer itself toward the future, we might find important insights into behavior.

Biology has not discouraged this article of faith. Anticipatory, context-sensitive pragmatism is a defining feature of life, even in brainless forms (Saigusa et al., 2008; Latty and Beekman, 2011). Anticipation suffuses popular neo-Darwinist understandings of evolution as life tailoring itself according to variable transmission of genotypes (see Brooks, 2005; Fodor and Piattelli-Palmarini, 2010; Griffiths and Gray, 2005; Lewontin, 1982; Smith, 2008 for widely ranging views for/against this interpretation). Life seems different, straddling a boundary between thermodynamics and information: thermodynamic provides the fuel, and information encodes biological particulars into signals guiding phenotypes away from dangerous future unknowns (Rosen, 1985). We never think of non-biological stuff interpreting anything, but if we lend biology powers of interpretation (Dennett, 1971), then systems steering themselves toward the future might seem less mysterious.

Cognitive science leaves biology to its own mysteries, but it extends the line of credit to the brain. Cartesian metaphors of the brain as seat of a soul-as-observer have been much maligned as question-begging. Everyone knows to swear off this ghostly homunculus (or perhaps, for a more modern era, “personcule”), but cognitive science still has had trouble explaining its faith in steering toward the future. We have learned from Lashley's (1950) futile search for specific cortical structures storing memories and Hebb's (1949) rephrasing of “representations” from contents of neural lockboxes to less offensive “fire-together/wire-together” patterns. Then again, we steer clear of vague holism blandly allowing that “Everything might do everything.” Cognitive science has grown confident carving brains and/or minds into modules, simple parts with domain-specific functions and evolutionary roots (Pinker, 1997). The trouble with this bounty of inherited modules is threefold: evolution does not know what purpose is Lewontin (1982), Fodor (2005) the genome carries much more genetic material than necessary to code for proteins while also leaving much of the hard work of building tissues to codeless, lifeless physics (Denton et al., 2003; Pearson, 2006); and brains do not support a stable anatomical organization respecting different cognitive, perceptual or behavioral domains (Graziano et al., 2002; Anderson, 2010; Hickok, 2014). This latter point requires spirited and repeated restatement against all-too-easy intuitions to the contrary.

Network theory was born of a hope that, one day, systems steering themselves toward future states might not require so much faith. Computer science, cybernetics and complex-systems theories raised fascinating new questions about the capacity of material systems to absorb sensory inputs and develop these sensory input into goal-directed behaviors (McCulloch and Pitts, 1943; Ashby, 1956). Turing (1936, 1937, 1950) led the vanguard in asking how intelligent machines could be. Some very simple machines did literally build themselves from dead material parts to choose sensors and learn rudimentary things about their surroundings (Pask, 1958). They were notoriously difficult to control and useless for our everyday purposes (Cariani, 1993). Some engineers suggested that orderly natural systems were built of modular parts and so that only modular systems were controllable ones (Simon, 1969)—and greater financial interest went toward building docile machines (e.g., for typing messages upon) than in breathing life into autonomous ones. Ultimately, these pioneering minds began to ponder whether intelligent behavior might build itself out of the busy connections among many non-intelligent component agents (Rosenblatt, 1958; Selfridge, 1959). This now-immense program of research began in fits and starts, always looking forward to the future possibility that the right interactive architecture might allow autonomous behavior steering toward the future (Minsky and Papert, 1969).

Like any newborn organisms learning to toddle around, some of these networks behaved more obediently than others. Some behaved well according to a supervisor's “delta rule” (Widrow and Hoff, 1960), dutifully computing upon sensory inputs and comparing its behavior with the engineer's feedback. Other networks were moodier and acted up on their own. Without any supervision, they began to amplify small fluctuations and share them among local parts, producing large, coherent behaviors that their designers had not planned. These unsupervised networks would not stop when they had an accurate or adaptive response. Instead, they kept generating new structures building on what they had done before or breaking it down. It is this latter, unruly sort of network that might be more interesting provided they ever learned anything. The former only acts to follow rules, but the latter has something close to creativity in its coordination, both attributes we find intriguing in biological systems.

These unruly networks clamber to their feet and generate their own large-scale behaviors using familiar methods: in the sand- or rice-pile network, single grains dropped at regular intervals pile up into dunes, and gradually, pile instability yields an avalanche, and one avalanche begets another (Jensen, 1998). We find similar avalanches in artificial neural networks built on similar principles. Neuronal avalanches appear as local field potentials in explicitly neural networks (Beggs, 2008). Both types of avalanches follow an inverse power-law structure consistent with “fractal” statistics: both probability density functions of avalanches and power spectra of avalanche time series' power spectrum decay slowly with increasing size or frequency, respectively. This slow decay entails that power laws never converge and never stop growing, suggesting that interactions among avalanches have a rich creativity bounded only by the size of the network (Bak et al., 1987). Of course, rice-piles are not brains and exhibit none of the intelligence that cognitive science seeks to explain (Wagenmakers et al., 2005). Novel work in a sort of neural network exhibiting what is called “critical branching” proposes to implicate the strength of power-law scaling of neural-spike trains with improved memory and computing capacity (Kello, 2013; Rodny and Kello, 2014). The self-organizing network is growing up and developing into an intelligent animal.

Power-law structure in self-organizing networks suggests that empirical evidence of power-law structure indicates interaction-driven, self-organizing processes (Bak, 1996; Van Orden et al., 2003; Friston et al., 2012). These proposals are more tantalizing in cognitive sciences than in “natural” ones, the latter being inured to the world's self-organization. Only in cognitive sciences does the glow of consciousness cast shadows on “natural-ness.” In the cognitive sciences, the rise of choice, goals, or forward-looking pragmatism make everything seem suddenly “not so natural” (Lewontin, 2010). Whatever “natural” is, cognitive science deals in behaviors driven by “agents,” “selves,” or whatever we call “personcular” ghosts. Agents stand apart from the self-organizing world beyond in their interpretive stance, browsing through reams of sensory codes to plot its leap forward into an imagined future, balancing nimbly between energy-burning process of collecting information and energy-conserving computations over that information (Brooks, 2005; Smith, 2008; Friston et al., 2012).

To iron out the ghosts lurking in this information-thermodynamics divide, we disregard any primitive distinction between conservable “information” and dissipated energy. Self-organizing networks give cognitive science two points of leverage. First, fractal statistics in empirical data from cognitive performance implicate similarly interactive architecture (Holden and Rajaraman, 2012; Abney et al., 2014). Fractal statistics are not simply concurrent with but outright predictive of cognitive performance (Stephen et al., 2009; Stephen and Hajnal, 2011). Second, given the overwhelmingly full-body evidence of fractal statistics beyond the confines of the skull (Hausdorff, 2007), network modeling allows us to clothe fractal systems with new theoretical elaborations.

A crucial point for theoretical development of networks is that our infant networks needed the “right” mix of constraints and unsupervised randomness to exhibit interesting self-organization (Beggs, 2008). Different interactive architectures leave networks free to discover different response patterns—just as task constraints can strengthen or diminish evidence of fractal structure (Kuznetsov and Wallot, 2011), it is possible to build a network that will not produce power-law-structured responses (Csányi and Szendröi, 2004). We might gradually tune control parameters to generate networks with gradually more fractal or less fractal architectures (Sporns, 2006), and we can use these parameters to describe the “connectome” of functional interactions spanning the brain (Zuo et al., 2012). Subtle changes in network topology in terms of connection strengths and connection patterns offers a rich testbed for creating novel hypotheses about development of perceiving-acting systems (Gorochowski et al., 2012; Ma et al., 2013). For instance, recent pioneering work in robotics has attempted to flesh out such neural networks, embedding networks into musculoskeletal architectures in order to model the dramatic effects of subtle factors such as uterine pressures and twitches during sleep on sensorimotor development (Blumberg et al., 2013; Mori and Kuniyoshi, 2013). In this way, network science has gradually blurred the lines between infant models and actual human infants.

Even without human-infant morphology, network modeling has brought its share of surprises. For instance, the sand/rice-pile model can fail to produce straightforward fractal fluctuations (Jensen et al., 1989). Even more stunning has been the evidence that the sand/rice-pile model may not just be fractal but, in fact, multifractal: it may exhibit several power-law forms at once (Tebaldi et al., 1999; Cernak, 2006; Bonachela and Muñoz, 2009), making it more complex. This multifractal wrinkle in the self-organization narrative may be exactly what's needed to help cognitive science play by more ordinary scientific rules. Observation of multifractal fluctuations offers the possibility that fractal fluctuations might interweave and spread into one another (Halsey et al., 1986). Where we might once have envisioned anatomical parts each with their own mysterious capacities, there may be less rigidly defined regions engaging in ongoing exchange of fractal and multifractal fluctuations.

The sharing of multifractal fluctuations has empirical anchoring in behaviors extending beyond the brain. Network analyses such as vector autoregression (VAR; Sims, 1980) allow us to depict the flow of information across nodes in full-body network, even from measurements of living, breathing organisms. For instance, infants' spontaneous leg kicking has been intuitively understood as an exploratory process, bringing “external” or “peripheral” information about gravity and leg kinematics “inward” to central nervous structures. However, VAR can elevate this intuition to empirically demonstrable fact: the flow of multifractal fluctuations along infants' legs from ankle to knee to hip does become a rigorously testable hypothesis (Stephen et al., 2012). Additionally, recent work in perceptual learning showed that use of visual feedback for a manual wielding task depends on time-varying fractal structure of head sway. Multifractality of head sway supports the pick up of visual information. VAR showed further that simply receiving visual feedback lets multifractality at the head spread down to the hand, thereby changing subsequent manual wielding (Kelty-Stephen and Dixon, 2014). The sharing of multifractal fluctuations may underwrite body-wide coordinations in ways that only network analyses have revealed.

Exciting as simulations may be, we see more promise in this latter attempt to draw from fractal statistics and matrix algebra to help us probe the full-body network. Distributing cognition across the body is still not repaying the loans of intelligence, but it may diminish the borrowed principal. Network modeling thus allows us to envision behavior—real, observed behavior—as the time-varying mixture of an extended field of multifractal fluctuations. Through this lens, behavior begins to require much less faith and much more like generic physical processes. Cognitive science need not ask to play by different rules or to start with different assumptions. On the contrary, network science might allow cognitive science operate on the same playing field as other sciences, whether sciences of living systems or otherwise.

## Conflict of interest statement

The authors declare that the research was conducted in the absence of any commercial or financial relationships that could be construed as a potential conflict of interest.
